# Classification of Microcalcification Clusters Using Bilateral Features Based on Graph Convolutional Network

**DOI:** 10.3389/fonc.2022.871662

**Published:** 2022-05-13

**Authors:** Yaqin Zhang, Jiayue Han, Binghui Chen, Lin Chang, Ting Song, Guanxiong Cai

**Affiliations:** ^1^ Department of Radiology, The Fifth Affiliated Hospital of Sun Yat-sen University, Zhuhai, China; ^2^ Department of Clinical Laboratory, Children’s Hospital of Nanjing Medical University, Nanjing, China; ^3^ Department of Radiology, The Third Affiliated Hospital of Guangzhou Medical University, Guangzhou, China; ^4^ School of Computer Science and Engineering, Sun Yat-sen University, Guangzhou, China

**Keywords:** breast cancer, microcalcification, graph convolutional network, computer-aided diagnosis, classification

## Abstract

Breast cancer is one of the diseases with the highest incidence and mortality among women in the world, which has posed a serious threat to women’s health. The appearance of clustered calcifications is one of the important signs of breast cancer, and thus how to classify clustered calcifications comes to be a key breakthrough in controlling breast cancer. In this study, the discriminant model based on image convolution is used to learn the image features related to the classification of clustered microcalcifications, and the graph convolutional network (GCN) based on topological graph is used to learn the spatial distribution characteristics of clustered microcalcifications. These two models are fused to obtain a complementary model of image information and spatial information. The results show that the performance of the fusion model proposed in this paper is obviously superior to that of the two classification models in the classification of clustered microcalcification.

## Introduction

Breast cancer is one of the diseases with the highest incidence and mortality among women in the world. According to the statistics of the World Health Organization, in 2020, there were 2.3 million new cases of breast cancer among women worldwide, and about 685,000 women died of breast cancer, accounting for 15.5% of all female deaths from malignant tumor (1). However, there is still a lack of detailed scientific understanding of the causes and mechanisms of breast cancer, and thus it is particularly difficult to prevent breast cancer (2). Therefore, early diagnosis and early treatment are particularly important for women with breast cancer. At present, as a relatively low-dose, safe, and low-cost means of image detection, all-digital mammography has become one of the best methods for routine clinical examination and preventive screening of breast cancer (3, 4).

The appearance of clustered calcifications is one of the important signs of breast cancer (5), and the high correlation between clustered calcifications and breast cancer has also attracted extensive attention in medical communities and academic circles. How to classify clustered calcifications comes to be an important breakthrough in controlling breast cancer.

In evaluating the possibility of malignant calcifications, the morphology and distribution of microcalcifications are equally important. The morphology of calcifications in breast cancer is an important factor to determine whether the calcifications are benign or malignant. Generally, according to the morphology, calcifications can be divided into being benign calcifications, intermediate concern calcifications, and calcifications with a higher probability of malignancy (6). The spatial distribution of microcalcifications in breast cancer is another important factor to distinguish between benign calcifications and malignant ones. The linear and segmental distributions are usually closely related to malignant calcifications, the diffused and regional distribution usually indicates benign calcifications, and the clustered distribution predicts intermediate concern calcifications (7). However, this classification is only a rough estimate based on experience, and the specific diagnosis still depends on needle biopsy.

In the research on the classification of clustered microcalcifications in mammography, most of the previous methods manually extract features of microcalcifications, then screen the features, and finally classify them by constructing a classifier.

Feature extraction and screening are the key to automatic classification of clustered microcalcification. Soltanian et al. (8) selected 15 characteristics in the cluster, including the number of microcalcifications, the maximum size of microcalcifications, the standard deviation of the size of calcifications, the number of calcifications with the size of 1 pixel, the total area of the microcalcifications, the average compactness, the maximum compactness (the ratio of the square of the perimeter to the area), the maximum moment representing the roughness of microcalcifications, the average roughness, the approximate circle radius, the scattering of the microcalcifications, the average gray level of the microcalcifications, the standard deviation of the mean of gray levels of microcalcifications, the maximum standard deviation of the gray levels of the calcifications, and the average standard deviation of the gray levels of the calcifications. Then, they trained the classifier to classify clustered calcifications as benign or malignant calcifications. Veldkamp et al. (9) used 16 features to classify microcalcifications, which can be split into two types, distribution features and morphologic features. These features comprise the distribution features of individual microcalcification in the cluster, the morphology of the cluster, and the position feature of the cluster. In mammography, the distribution features include the number of calcifications in the cluster, as well as the mean and standard deviation of pixels, direction, contrast, eccentricity, and compactness of microcalcifications. Moreover, the morphologic features of microcalcification clusters contain the area, the eccentricity, and the orientation of calcification clusters. Furthermore, the position features of clustered calcifications mainly refer to the relative distance between clustered calcifications and pectoralis major as well as the relative distance between clustered calcifications and the breast margin.

At the same time, the classifier plays an important role in the computer-aided diagnosis of microcalcifications in mammography. The classifier is trained by using these extracted features or a screened subset of features, so as to classify microcalcifications as benign or malignant microcalcifications. Lee et al. (10) used the artificial neural network based on shape recognition, which has a general shape feature layer and can extract general rules by learning from examples. The evaluation of the system by Nijmegen mammography database shows that its sensitivity and specificity can reach 86.1% and 74.1%, respectively. Ferreira et al. (11) designed a nearest neighbor classifier, which used Euclidean distance as the metric between the corresponding wavelet coefficients to verify the classification. Veldkamp et al. (9) used the classification method of clustered calcifications based on k-Nearest Neighbor (KNN), which firstly assigned a benign or malignant probability value to each clustered calcification and then averaged the probability values of clustered calcification in Cranio Caudial (CC) view and mediolateral oblique (MLO) view of patients as the final benign or malignant predictive value.

However, all of these methods have two common defects. On the one hand, it is necessary to manually predefine the lesion area of clustered microcalcifications, so as to characterize features and extract the features of microcalcifications and clusters. This process is cumbersome, and its labor cost is high. On the other hand, the feature space constructed by traditional methods often needs to extract the morphologic features of microcalcifications. However, because of weak signals and noise, the features of microcalcifications are difficult to exact, including morphological features and texture features. Hence, it is a challenging task to study how to build an effective and robust classification model.

Deep convolutional neural network has been widely used in image classification, image recognition, natural language processing, and other fields and has attracted the attention of academic circles in recent years (12–14). The main advantage of deep learning method used for classification of microcalcifications in breast cancer lies in that it can directly learn features and patterns related to benign or malignant classification from a large number of microcalcifications data through supervised learning, without manually constructing and screening corresponding classification features for microcalcifications (15).

Inspired by the clinical diagnosis mechanism, this study also sets out from the morphology and distribution of microcalcifications and constructs a model to distinguish benign microcalcifications from malignant ones. Firstly, with regard to the morphologic features of microcalcifications, this study uses the deep convolutional neural network to extract hidden layer features related to classification in images. For the spatial distribution features of microcalcifications, the artificially constructed features mainly include the number of calcifications in the cluster, the mean and standard deviation of pixel direction, contrast, eccentricity and compactness of microcalcifications, and so on, which are difficult to extract by general convolutional neural network. In order to solve this problem, this study constructs a topological graph model and uses the graph convolutional neural network to learn the spatial distribution features of microcalcifications in the breast. Then, the above two models are fused through the modal fusion and voting strategy, and finally the benign or malignant predictive value of the whole input image is the output.

## Materials and Methods

### Data Set

**Figure 1:**
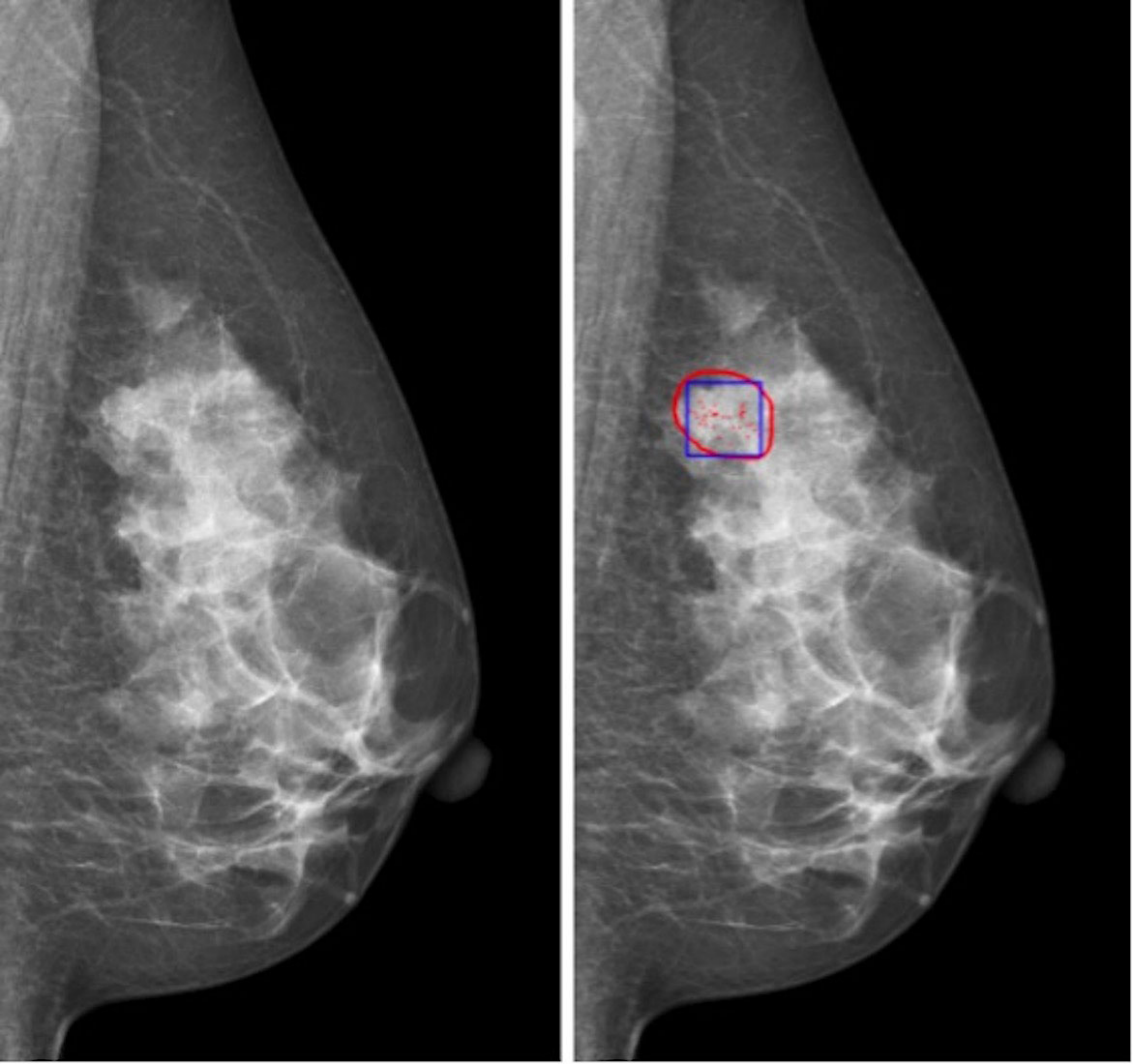
Region of interest of microcalcification cluster for classification: (left) original image; (right) red contour is the possible region of interest of microcalcification, and blue box is the region of interest of microcalcification cluster for classification.

The data set of this study is from Sun Yat-sen University Affiliated Hospital, which contains the data of 197 cases (394 two-dimensional mammograms). The approval of the institutional review board (IRB) had been obtained before the data set was collected. The acquisition equipment is Mammo-Novation Siemens imaging equipment and solid-state detector of amorphous selenium with a pixel space size of 70 μm/pixel and a resolution of 2,560 × 3,328 pixels or 3,328 × 4,084 pixels. All cases included two kinds of mammograms, namely, axial view and MLO view. The results of all cases are confirmed by biopsy and hence can be used to evaluate the effectiveness of the diagnostic system proposed. An experienced radiologist marked a rectangular lesion area of clustered microcalcifications and the corresponding results of biopsy (**Figure 1**). Then, the labels were confirmed and revised by another experienced radiologist as the gold standard of the final experiment. The data used in this study are selected cases that only contain microcalcifications; that is, they do not contain lumps, structural distortion, and other diseases. Therefore, the benign or malignant results obtained by needle biopsy can only reflect whether microcalcifications are benign or malignant, so the gold standard meets the requirements without ambiguity.

### Methods

#### Discriminant Model Based on Spatial Convolution Network

In the classification task of benign or malignant clustered microcalcifications, the regions of interest are irregular with different sizes. The large one may occupy the whole or half of the breast, while the small one may only spread in the area with a diameter of a dozen pixels. Therefore, it is not realistic to use a unified and large frame to capture the lesion area and input it into the network training. Especially for small lesions, too large a frame may cause the lesion area to occupy only a small part of the captured image. However, it is not appropriate to use image scaling because the microcalcifications in the image are so small that their area is only a dozen pixels. With image scaling, a large amount of microcalcification lesion information may be lost.

**Figure 2:**
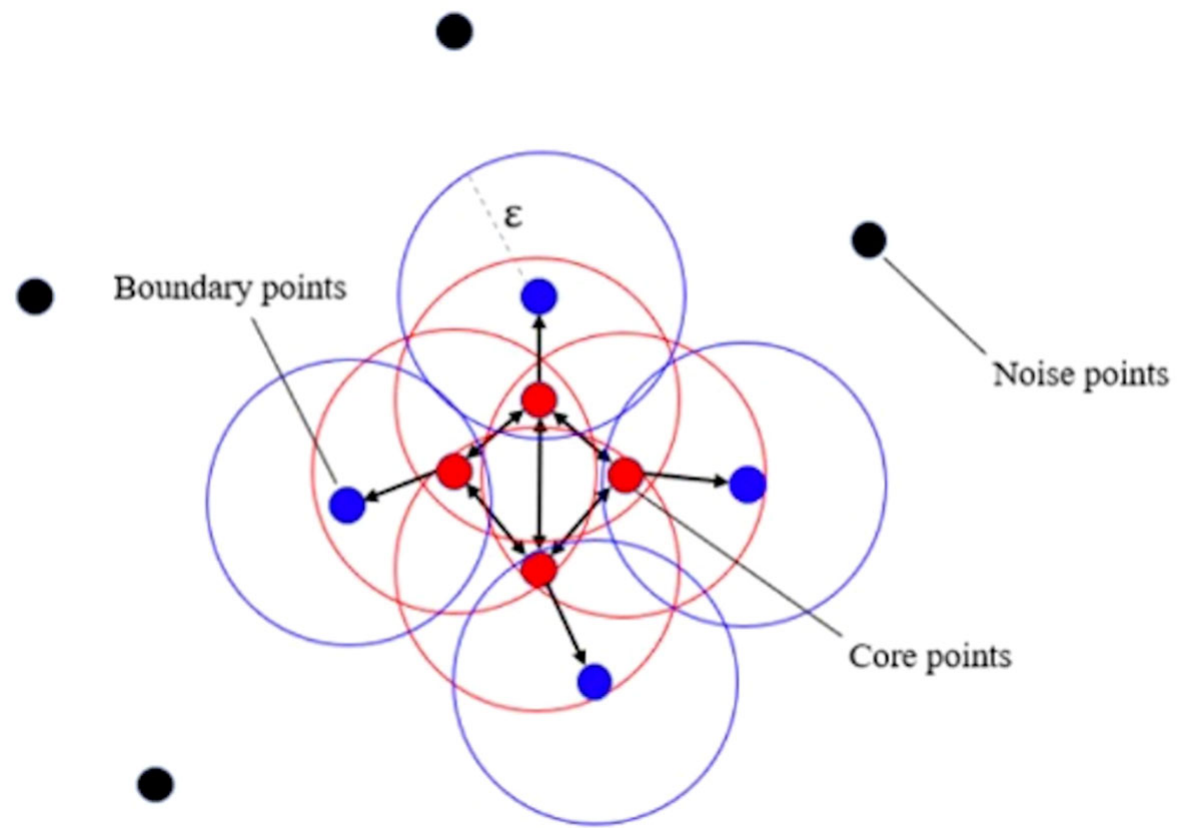
Illustration of density-based spatial clustering of applications with noise algorithm.

In order to solve this problem, this study adopts the strategy of splitting the large cluster using clustering algorithms. We adopted a density-based spatial clustering of applications with noise algorithm (11) (see **Figure 2**). By adjusting the cluster radius, the cluster is prevented from forming too large clusters, so that the originally large clusters will be split into several subclusters with appropriate sizes. In this circumstance, the researcher can take these subclusters as the center, use a frame with a uniform size to capture the lesion area, and input the image into the network for training. The strategy of splitting large clusters can be applied to benign cases, while it is not suitable for malignant cases. Because in malignant cases, it is likely that only part of the large clusters is malignant, while other areas are still benign. However, the needle biopsy only obtains the gold standard of the whole case and cannot accurately locate the specific malignant lesions in large clusters. Therefore, for malignant cases, only some cases without particularly large clusters can be selected for training, so that the frame with a uniform size is still applicable here. Malignant cases with relatively large clusters are classified into a verification set and a test set.

**Figure 3:**
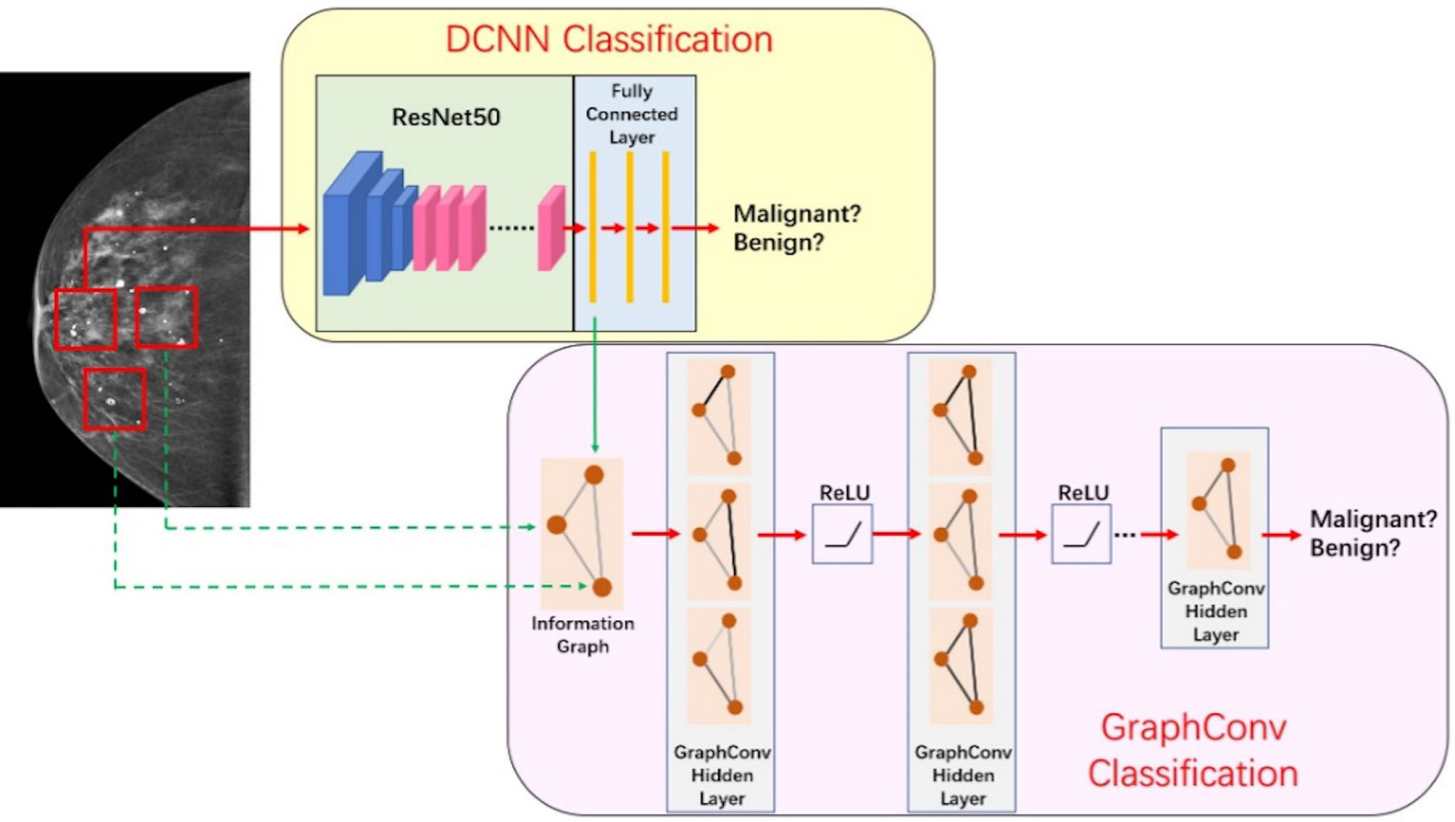
Illustration of the network structure used for classification.

The discriminant network based on spatial convolution mainly adopts the deep convolutional neural network based on ResNet-50 (12) structure (see **Figure 3**), which conducts multilayer perceptual learning through convolution, downsampling, and nonlinear activation, and uses the back propagation and stochastic gradient descent algorithm to seek the parameters of the network model. In the training process, low-layer features will constitute high-layer features through automatic composition, and finally features will be screened and classified by a linear model. The size of the input image is 3 × 224 × 224, and the output is a benign or malignant predictive value between [0,1]. Because there is only one channel in the gray image, the other two channels are filled by replication method to form the 3-channel network input layer.

**Figure 4:**
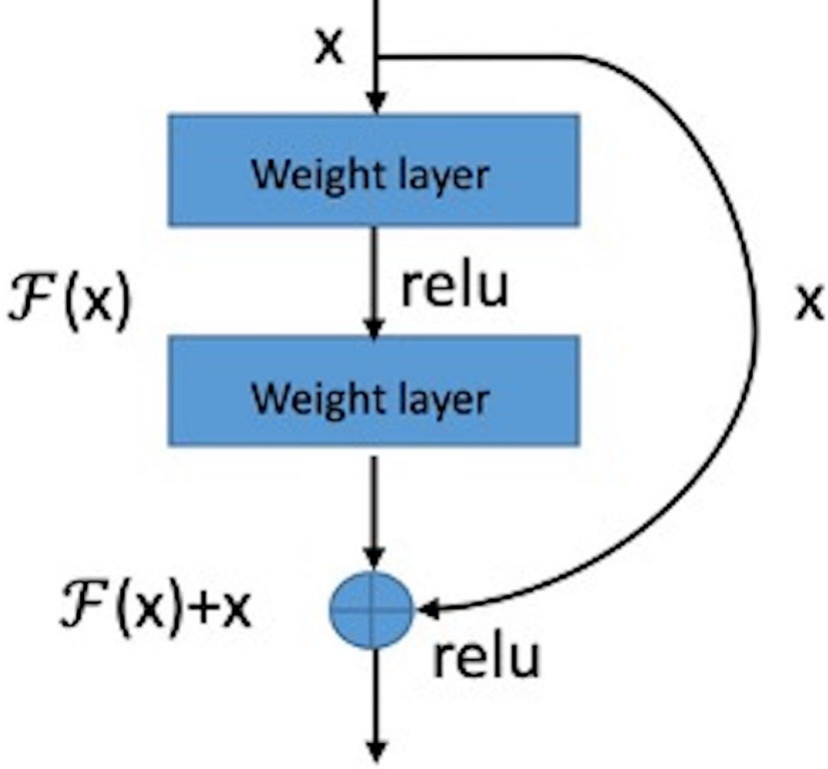
Structure of a residual block.

The basic structure module of the residual network is shown in **Figure 4**. The structure of the feed-forward non-residual network is mostly y = H(x), where x and y are input and output of the residue block, respectively. The residual block of the residual network can be expressed as H(x) = F(x) + x, that is F(x)= H(x) – x. Consequently, the network learns the residual of input variables, which is equivalent to a differential amplifier. It is difficult to learn microcalcifications directly because of its small size and low contrast. But using a residual can make the network pay more attention to the details of microcalcifications, so it is a better solution.

#### Discriminant Model Based on the Graph Convolutional Network

**Figure 5:**
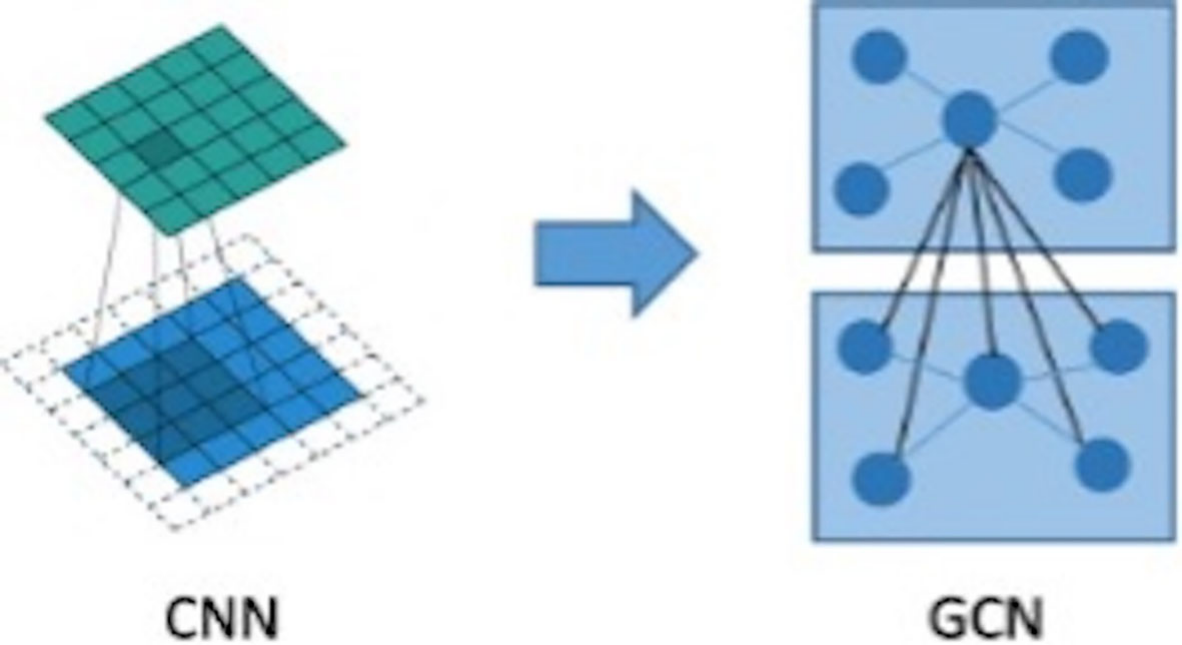
Generalization from image spatial convolution to graph convolutional network.

The graph convolutional network (GCN) can extract spatial distribution. The convolutional neural network studies the statistical characteristics of Euclidean data with a regular spatial structure, such as image, speech sounds, text sequence, and so on. Essentially, a convolution in the convolutional neural network uses a filter with shared parameters and constructs a feature map by calculating the weighted sum of pixel values of the center point and adjacent points so as to extract image features (16). However, in this research, microcalcifications have rather complicated spatial laws and do not have neatly arranged pixel elements like image matrix, which means the spatial position relationship between microcalcifications is non- Euclidean, and image convolution may be difficult to extract image features for microcalcification clusters. In order to extract spatial distribution features between clustered microcalcifications through the network, this study firstly constructed the relationship between microcalcifications as a graph model, in which the nodes are hidden layer features of convolutional neural network of microcalcifications and the edges are Euclidean distances between central pixels of microcalcifications. Then, what this study needs to solve is how to choose a fixed convolution kernel to adapt to the irregularity of the whole graph and thereby construct a feed-forward network to extract spatial distribution features from node information and edge information. It means that this study should construct a graph convolution operation similar to the extension of image convolution on a topological graph, as shown in **Figure 5**. The starting point of convolution on a topological graph is similar to that of image convolution. The basic idea is to generate a new feature graph by integrating the features of the points adjacent to the central point and parameterize the convolution kernel to attain an optimized solution by building a network (16–18).

The constructed graph is recorded as *G* = (*V*, *E*), the element in *V* is the vertex of the graph (microcalcifications), and the element in *E* is the side between the vertices. The neighbors of vertex *v_I_* are defined as:


(1) $$\left(i)=\{v_{j}\in V|v_{i}v_{j}\in E\right\}$$

The degree matrix is a diagonal matrix describing the degree of each vertex *v_i_*, namely, (*v_i_*):


(2) $$D(G)=\left(\begin{array}{ccc} d(v_{1}) & \cdots & 0\\ \vdots & \ddots & \vdots \\ 0 & \cdots & d(v_{n}) \end{array}\right)$$

Adjacency matrix is an n-order square matrix describing the spatial position relationship between vertices. It mainly encodes the spatial distribution information of the graph network, which is defined as:


(3) $${\left[A(G)\right]}_{ij}=\{\begin{array}{ll} dist_{ij} & if\;v_{i}v_{j}\in E\\ 0 & otherwise \end{array}$$

Laplacian matrix, also known as admittance matrix (17), is mainly used in graph theory. For graph *G* = (*V*, *E*), the Laplace matrix is defined as the difference between the degree matrix *D* and the adjacency matrix *A* of graph *G*:


(4) $$L=D-A=\{\begin{array}{cccc} d(v_{i}) & & & if\;i=j\\ -dist_{ij} & & & if\;\neq \;j\;and\;v_{i}v_{j}\;\in \;E\\ 0 & & & otherwise \end{array}$$

Obviously, the Laplace matrix*L*is a symmetric matrix. In fact, it is easy to prove that it is a positive semidefinite matrix; that is, the quadratic form is greater than or equal to 0. Usually, the experiment will normalize it, so the symmetric normalized Laplace matrix is obtained:


(5) $$\tilde{L}=D^{-\frac{1}{2}}LD^{-\frac{1}{2}}=I-D^{-\frac{1}{2}}AD^{-\frac{1}{2}}$$

Considering that the calculation of convolution in the frequency domain is relatively simple, the Fourier transform on the graph is introduced to construct the convolution on the topological graph. Taking the feature vectors of Laplace matrix as the basis of Fourier transform on the graph, the following is obtained:


(6) $$F(\lambda_{l})=\hat{f}(\lambda_{l})=\sum_{i=1}^{n}f(i)u_{l}^{*}(i)$$

The following is obtained when it is expressed in matrix form:


(7) $$\left(\begin{array}{c} \hat{f}(\lambda_{1})\\ \vdots \\ \hat{f}(\lambda_{N}) \end{array}\right)=\left(\begin{array}{ccc} u_{1}(1) & \cdots & u_{1}(N)\\ \vdots & \ddots & \vdots \\ u_{N}(1) & \cdots & u_{N}(N) \end{array}\right)\left(\begin{array}{c} f(1)\\ \vdots \\ f(N) \end{array}\right)$$

Also


(8) $$U^{T}=\left(\begin{array}{ccc} u_{1}(1) & \cdots & u_{1}(N)\\ \vdots & \ddots & \vdots \\ u_{N}(1) & \cdots & u_{N}(N) \end{array}\right)$$

Then, the signal *f* of Fourier transform on the graph is


(9) $$\hat{f}=U^{T}f.$$

Inverse transform to be


(10) $$f=U\hat{f}$$

According to the convolution theorem, the Fourier transform of function convolution is equal to the product of function Fourier transform:


(11) $$f*h=F^{-1}\left[\hat{f}(\omega )\hat{h}(\omega )\right]=\frac{1}{2\pi }\int \hat{f}(\omega )\hat{h}(\omega )e^{i\omega t}d\omega$$

The convolution operation on a graph can be derived; that is, graph convolution (16):


(12) $${\left(f*h\right)}_{G}=U((U^{T}h)⊙(U^{T}f))=U\cdot diag\left(\hat{h}(\lambda )\right)\cdot U^{T}f$$

However, when the above convolution is actually used, there exists the following difficulties in solving the convolution kernel. First of all, the product of *U*, *diag* $\left(\hat{h}(\lambda )\right)$ and *U^T^* needs to be calculated in each forward propagation with a complexity of (*n* ^2^). Besides, the convolution kernel has n parameters and does not have spatial locality.

In order to solve the above problems, the Chebyshev polynomial k-order truncation is used to approximate the $diag\hat{h}(\lambda ))$ ,


(13) $$h_{\theta }(\Lambda )\approx \sum_{\text{k}=0}^{\text{K}}{\theta^{\prime }}_{k}T_{k}\left(\tilde{\Lambda }\right)$$

(14) $$\tilde{\Lambda }=\frac{2}{\lambda_{\max}}\Lambda -I_{N}$$

So,


(15) $${\left(h_{\theta }*x\right)}_{G}\approx \sum_{\text{k}=0}^{\text{K}}{\theta^{\prime }}_{k}T_{k}\left(\tilde{L}\right)x.$$

(16) $$\tilde{L}=\frac{2}{\lambda_{\max}}L-I_{N}$$

At this time, the graph convolution does not depend on the whole graph but only on the k-order neighbors of the current central node.

The GCN is constructed. Although the above k-order approximation can establish the dependence of k-order neighbors, it still needs to perform k-order operation on L. In order to further reduce the calculation process, k is limited to 1. At this time, the graph convolution can be approximated as a linear function of L:


(17) $${\left(g_{\theta }*x\right)}_{G}\approx {\theta^{\prime }}_{0}x+{\theta^{\prime }}_{1}(L-I_{N})x={\theta^{\prime }}_{0}x+{\theta^{\prime }}_{1}D^{-1/2}AD^{-1/2}$$

There are only two shared parameters to be trained in the above formula. To establish k-order neighbor dependence, k-th continuous first-order graph convolution operation can be adopted to construct forward propagation:


(18) $$Z=f(X,A)=softmax(\tilde{A}ReLU(\tilde{A}XM^{\left(0\right)})W^{\left(1\right)})$$

The loss function adopts the cross-entropy loss function:


(19) $$L_{ce}=-\frac{1}{N}\sum_{i=1}^{N}\sum_{j=0}^{nK}y_{ij}\text{ log}p_{ij}$$

#### Fusion of Discriminant Model

The discriminant network based on spatial convolution is mainly used to extract the image features of clustered microcalcifications, including morphologic features. The discriminant model based on the graph convolution is mainly used to extract the spatial distribution characteristics of microcalcifications. They need to be fused to make full use of the extracted image information and spatial distribution information, so as to comprehensively diagnose the input microcalcifications images as benign or malignant.

**Figure 6:**
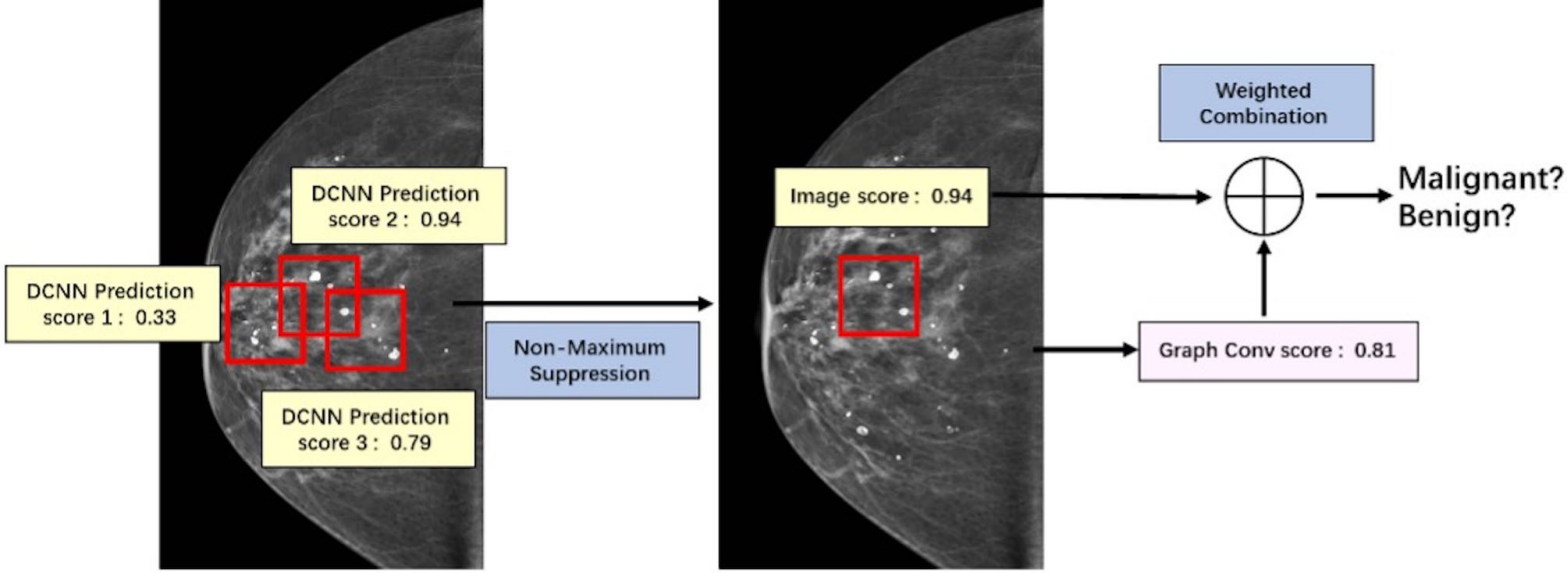
Illustration of fusion process of discriminant model.

The key of this part is how to fuse the extracted image information and spatial distribution information. Image information and spatial distribution information can be regarded as two different modes, so the problem comes down to multimodal fusion. There are many ways to solve multimodal fusion, such as element-by-element weighted summation, element-by-element maximum pooling, gated activation, gated attention, bilinear mapping, and so on (19). According to different levels, it can be fused at the feature level, such as splicing, adding, and so on, at the score level, such as weighting based on the scores of different modes obtained from training, and at the decision-making level, such as majority voting, maximum voting, and so on. First, this study used non-maximum suppression in the scores obtained from the spatial convolution discriminant model to obtain the predictive scores based on images at the spatial convolution level. Second, this study combined the predictive scores with the scores obtained after the topological graph convolution extracted the spatial distribution information. Last, using the fusion method at the score level and the corresponding weights of image information mode and spatial distribution information mode obtained through training, the scores are weighted and fused at the full connection layer (20) to output a final benign or malignant predictive value. The fusion process of discriminant model was illustrated in **Figure 6**.

#### Training of Benign or Malignant Classification Network

The discriminant network based on the spatial convolution adopts the convolutional neural network based on the ResNet-50 structure. The size of the input image is 3 × 224 × 224, and the output is a benign or malignant predictive value between [0,1]. Because there is only one channel in the gray image, the other two channels are filled by the replication method to form the 3-channel network input layer.

There were 273 benign samples and 273 malignant samples in the testing data set. The training, validation and testing data sets were separated as shown in **Table 1**. During training, data augmentation was carried out on the samples in the training set, mainly by random rotation, adding Gaussian noise, and so on, to expand the training samples. After that, the network can learn some more essential and stable features, and thus the trained discriminant model is more robust.

**Table 1:** Number of samples for training, verification, and testing in benign or malignant.

	Patch Numbers		
	Training set	Validation set	Test set
**Benign**	590	227	273
**Malignant**	831	227	273

**Figure 7:**
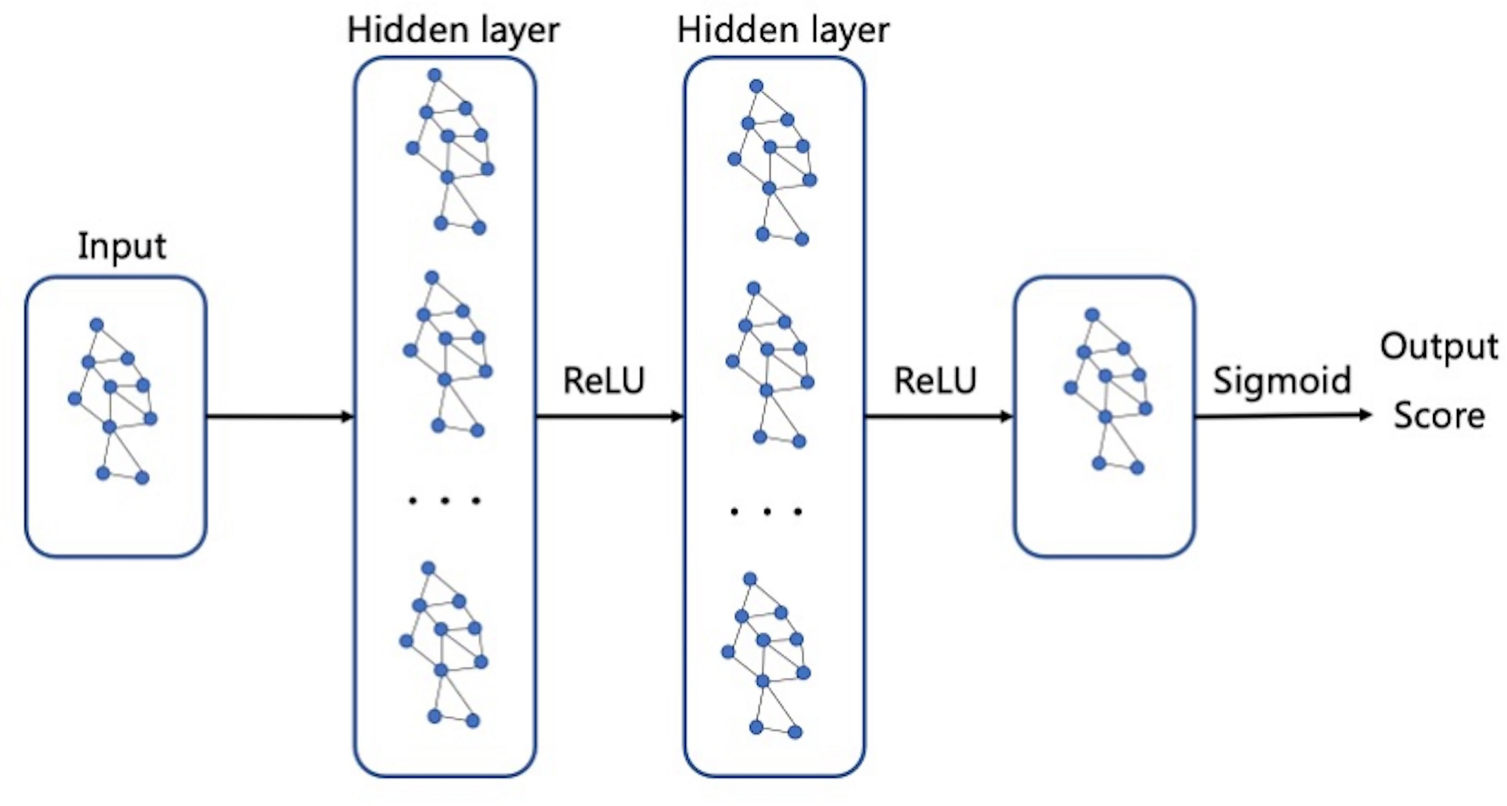
Graph neural network.

The discriminant network based on the spatial convolution adopts the graph convolutional neural network, as shown in **Figure 7**. The input is the topological graph composed of microcalcifications. The feed-forward network contains two hidden layers, among which the rectified linear unit (ReLU) activation function (14) is used. After the input goes through the feed-forward network and the action of Sigmoid activation function (21), the benign or malignant predictive values between [0,1] are output finally. Back propagation adopts the two-class cross entropy loss function, and the stochastic gradient descent optimizer is used for optimization.

## Results

### Establishing Quantitative Evaluation Indicators

In order to more clearly compare the results of different methods in the classification of benign or malignant clustered microcalcifications, this study uses sensitivity, specificity, and receiver operating characteristic (ROC) curve as relevant quantitative evaluation indexes to evaluate the classification results of clustered microcalcifications in images.

### Comparison of Results

**Figure 8:**
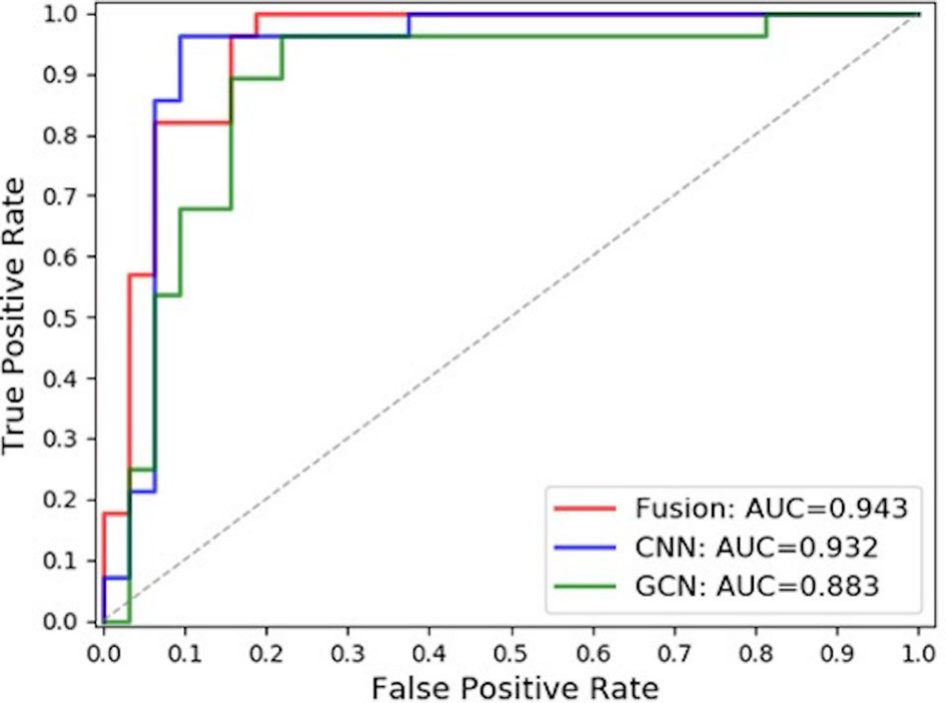
ROC curve comparison.

In this paper, the comparison was made among the test results of the discriminant network (referred to as ResNet-50) based on the spatial image convolution to extract the image information related to the classification of benign or malignant subclusters, those of the GCN based on the topological graph convolution to extract the spatial distribution information of microcalcifications related to the classification of benign or malignant clustered microcalcifications and those of the fused network (referred to as ResNet50-GCN Fusion). The results are shown in **Table 2** and **Figure 8**.

**Table 2:** Comparison of classification results of clustered microcalcification in different methods.

Methods	TPR	TNR	AUC
ResNet50	0.964	0.906	0.932
GCN	0.904	0.782	0.883
ResNet50-GCN Fusion	1.000	0.812	0.943

## Discussion

The classification of benign or malignant clustered microcalcifications in breast cancer mainly considers the morphology and spatial distribution of microcalcifications clinically. Taking this as the starting point, this study proposed to use the discriminant model based on image convolution to learn the image features related to the classification of microcalcifications and use the GCN based on the topological graph to learn the spatial distribution features of microcalcifications. After that, this study tried to fuse them to get a complementary model.

In this study, the model based on the spatial image convolution obviously performed better than the model based on the topological graph convolution both in sensitivity and specificity. The AUC area under the ROC curve of the former model is also nearly 5% higher than that of the latter. This shows that the image information for the diagnosis of benign or malignant clustered microcalcifications learned by the model based on spatial image convolution is very helpful for classification to a certain extent. Although the overall result of the model based on the topological image convolution is not as good as that based on the spatial image convolution, the spatial distribution information extracted by the model is still effective to a certain extent. Especially, if this distribution information has certain orthogonality with the image information, it will contribute more and the researcher can make use of the two information to combine their advantages and obtain a stronger classification fused model.

In fact, from the comparison results in the table, the AUC area under the ROC curve of the fused model reaches 0.943, which is about 1% higher than the AUC of ResNet-50 of the spatial image convolution, the best model in single mode. This shows that the spatial distribution information extracted by the GCN model based on the topological graph convolution exerts a complement action. However, it is worth noting that when the topological graph was input into the construction of the GCN in this paper, the features of the nodes of the graph were the hidden layer features of false-positive identification of microcalcifications, which actually limited the classification ability of the GCN to a certain extent. If the features related to the benign or malignant microcalcifications can be obtained, it is natural to guess that the overall ability of the GCN to classify the benign or malignant microcalcifications will be better. But this is exactly the difficulty of the research. In this study, it is difficult to obtain the mark of benign or malignant microcalcifications because doctor’s labeling is costly and highly subjective. Moreover, it is unrealistic to perform needle biopsy and registration for each microcalcification. Furthermore, previous research has never involved this area. Therefore, the following research can further improve the model to replace the features of nodes in the GCN with those of diagnosis of benign or malignant microcalcification if the labeling information of benign or malignant microcalcifications is obtained. It may be easier to capture the hidden features of the classification of benign or malignant microcalcifications and their spatial distribution law in the breast, which will be more effective for the results.

When constructing the graph network, the node features used in this study are the hidden layer features of the false-positive discrimination network of microcalcification detection. If the benign and malignant labeling information at the microcalcification level can be obtained in the subsequent research, the model can be further improved to replace the features of the GCN nodes with the features of microcalcification benign and malignant discrimination level. It may be easier to capture the differences between benign and malignant microcalcifications and their spatial distribution in the breast, which may be more helpful to the results.

## Conclusion

There are some obstacles in the classification of benign or malignant clustered microcalcifications in mammograms. In this study, image information and spatial distribution information are modeled on the issue of the classification of benign or malignant microcalcifications in breast cancer. The discriminant network based on spatial image convolution is constructed to extract the image information related to the classification of microcalcifications subclusters, and the discriminant network based on the topological graph convolution is proposed and constructed to extract the spatial distribution information of microcalcifications related to the classification of benign or malignant clustered microcalcifications. This study used non-maximum suppression in the scores obtained from the spatial convolution discriminant model to obtain the predictive scores based on images at the spatial convolution level. Then, this study combined the predictive scores with the scores obtained after the topological graph convolution extracted the spatial distribution information. Lastly, using the fusion method at the score level and the corresponding weights of image information mode and spatial distribution information mode obtained through training, the scores are weighted and fused to output a final benign or malignant predictive value. The results show that compared with the single-mode classification results, the classification after modal fusion is more accurate. Automatic detection and classification of microcalcification clusters may have an important impact in breast cancer screening.

## Data Availability Statement

The raw data supporting the conclusions of this article will be made available by the authors without undue reservation.

## Ethics Statement

The studies involving human participants were reviewed and approved by Sun Yat-sen University Affiliated Hospital. Written informed consent for participation was not required for this study in accordance with the national legislation and the institutional requirements.

## Author Contributions

YZ, GC, and LC contributed to conception and design of the study. YZ organized the database. LC performed the statistical analysis. TS helped to review the labels of microcalcifications. GC wrote the first draft of the article. JH and BC wrote sections of the article. All authors contributed to article revision and read and approved the submitted version.

## Funding

This work was supported by the National Natural Science Foundation of China (81801809) and the Basic and Applied Basic Research Foundation of Guangdong Province (2020A1515010572).

## Conflict of Interest

The authors declare that the research was conducted in the absence of any commercial or financial relationships that could be construed as a potential conflict of interest.

## Publisher’s Note

All claims expressed in this article are solely those of the authors and do not necessarily represent those of their affiliated organizations, or those of the publisher, the editors and the reviewers. Any product that may be evaluated in this article, or claim that may be made by its manufacturer, is not guaranteed or endorsed by the publisher.
